# Improvements in Undergraduate Oncology Education Introduced at Polish Medical Universities Between 2004 and 2010 Under Poland’s “National Program for Combating Neoplastic Diseases”

**DOI:** 10.1007/s13187-014-0633-y

**Published:** 2014-03-15

**Authors:** Rafał Matkowski, Jolanta Szelachowska, Krzysztof Szewczyk, Urszula Staszek-Szewczyk, Jan Kornafel

**Affiliations:** Department of Oncology and Division of Oncological Surgery, Wroclaw Medical University, Plac Hirszfelda 12, 53-413 Wroclaw, Poland

**Keywords:** Oncology, Undergraduate medical education, Curriculum, Poland

## Abstract

Cancer patient treatment in Poland remains unsatisfactory when compared to that in other countries. In 2005, this alarming situation prompted the Polish government to launch the “National Program for Combating Neoplastic Diseases” (NPCND). One part of this project was to improve the quality of oncology instruction at the undergraduate level over the years 2006 and 2007 (subsequently extended until 2010 thanks to promising results and the relatively small financial outlay). The program’s main aims were to improve existing oncology therapy and to ameliorate the quality of undergraduate oncology education. To evaluate the changes in the quality of undergraduate education as a result of the NPCND program, medical universities were asked to fill out a questionnaire. Responses indicate that the program had a major positive impact on the quality of cancer education mainly as a result of the introduction of a uniform program of training and an increase in the number of classes devoted to oncology. The main unresolved problem is that university hospitals seldom have integrated units catering in-house for surgery, radiotherapy, chemotherapy, etc., and most such “hands-on” teaching still has to be done externally.

## Introduction

The results of cancer patient treatment in Poland remain unsatisfactory. According to EUROCARE reports, Polish oncology patients have a much lower chance of surviving 5 years after diagnosis than elsewhere in Europe [1, 2]. This problem, which undoubtedly reflects Poland’s low healthcare budget [3], has for several years stirred lively debate in Poland. The main causes identified were the low health awareness of Polish society at large, the inadequate education of physicians [4–6], lack of funds for delivering healthcare, and the small number of dedicated oncology centers. To improve the situation, on July 1, 2005, the Polish government launched the “National Program for Combating Neoplastic Diseases” (NPCND). The program’s principal aims were to introduce free public screening for breast, cervical, and colorectal cancers, as well as to procure investment in Polish oncology centers in order to provide equipment for radiotherapy and advanced tumor diagnosis. Additionally, one part of the program coordinated by Wroclaw Medical University and implemented from 2006 was to improve the quality of oncological instruction at the undergraduate level.

## Material and Methods

Under the NPCND program launched in 2005, short-term financial and organizational supports were planned for the years 2006–2007 to improve the quality of undergraduate oncology education at all medical universities. After promising results in the first 2 years and recognizing the continued importance of the problem, the NPCND board decided to extend financial support until 2010.

The Polish academic oncology community has determined [4, 6, 7] that the most important tools for improving undergraduate oncology education in Poland include the following:

- Development of integrated oncology institutions (radiotherapy + chemotherapy + oncologic surgery + oncologic gynecology) within the structures of medical universities
- Building and maintaining a well-trained multidisciplinary staff of academic teachers at each medical university
- Introduction of a uniform curriculum complying with European Union recommendations
- Appointment of an oncology education coordinator at each medical university
- Preparing an oncology textbook for students
- Introduction of a uniform oncology final examination test at all medical universities.

To evaluate the hoped-for improvements in undergraduate training standards, oncology teachers at 11 Polish medical universities were asked to fill out a detailed questionnaire. Staff at eight universities responded to the request between December 2009 and May 2010. A range of questions were asked, ranging from the appointment of oncology education coordinators, available training facilities and organization of tuition, duration of classes, numbers and types of teaching staff, manner of instruction in palliative medicine, and finally to the main remaining problems associated with oncology teaching. The responses received were then compared with data reflecting the state of affairs in 2004, gathered in an almost identical questionnaire back in 2004 when a report on the state of undergraduate education was being prepared. A summary of responses to the questionnaire are provided in Tables 1, 2, and 3.

**Table 1:** Responses to questionnaire

Medical university (MU)	Separate course on oncology in the final years of studies		Introductory course on oncology (propedeutics in oncology) in the first years of studies		Hours of education in palliative medicine	
	Number of hours per year					
	2004	2010	2004	2010	2004	2010
MU Bialystok	45	60	0	15	2—part of oncology course	11—part of oncology course
MU Bydgoszcz	45	75	0	30	30—separate course	30—separate course
MU Gdansk	79	70	10	15	30—separate course	30—separate course
MU Lublin	50	60	0	0	4—part of oncology course	9—part of oncology course
MU Lodz	60	60	18	18	2-day classes—part of oncology course	3-day classes—separate course
MU Silesia	30	45	0	15	Separate course	35—separate course
Pomeranian MU	30	45	10	15	24—separate course	24—separate course
Wroclaw MU	40	60	0	20	12—separate course	12—separate course

Questions referring to a separate course in oncology in the final stage of studies, an introductory course in oncology (propedeutics in oncology) in the first years of studies, and education in palliative medicine

**Table 2:** Responses to questionnaire

Medical university (MU)	Is there a university coordinator of oncology education		Is there an examination on completion of oncology training		Education facilities: YES, provided by MU; NO, provided by an external oncology hospital		
	Year						Outside university
	2004	2010	2004	2010	2004	2010	
MU Bialystok	No	Yes	No	Yes	No	Yes	S, RT
MU Bydgoszcz	No	Yes	Yes	No	No	No	S, RT, CT
MU Gdansk	No	Yes	Yes	Yes	Yes	Yes	–
MU Lublin	No	Yes	No	No	No	Yes	RT
MU Lodz	No	Yes	Yes	Yes	No	No	S, RT, CT
MU Silesia	Yes	Yes	No	No	Yes	Yes	RT
Pomeranian MU	Yes	Yes	Yes	Yes	Yes	Yes	RT
Wroclaw MU	Yes	Yes	Yes	Yes	No	No	S, RT, CT

Questions referring to appointment of a university coordinator of oncology education, final examination to complete oncology training, and training facilities (whether provided by the university or an external oncology hospital)

*S* surgery, *RT* radiotherapy, *CT* chemotherapy

**Table 3:** Responses to questionnaire

Medical university (MU)	Academic teaching staff in the four basic oncology specialties: radiotherapy, chemotherapy, oncological surgery, and oncological gynecology			Problems considered most significant at the individual university
	Number of oncology teachers in year		Lack of	
	2004	2010		
MU Bialystok	6	7	Oncological gynecologist	No separate palliative medicine course, low number of teachers
MU Bydgoszcz	11	11	Oncological gynecologist	No final exam; inadequate lecture rooms and classrooms
MU Gdansk	15	10	Oncological gynecologist, oncological surgeon	Too few lecture rooms
MU Lublin	8	8	Oncological gynecologist	–
MU Lodz	21	22	Oncological gynecologist	–
MU Silesia	>8	>20	Radiotherapist	No exam; lack of teachers—radiotherapist
Pomeranian MU	15	20	–	Inadequate facilities; radiotherapy outside of university
Wroclaw MU	15	15	–	Inadequate lecture rooms and classrooms, radiotherapy outside of university

Questions referring to numbers of teaching staff in the four basic oncology specialties and problems considered most significant at individual universities

## Results

### Program Achievements

The most important achievements of the NPCND program between 2006 and 2010 are listed below.

1. Programs of education in oncology were developed at Wroclaw Medical University’s Faculty of Medicine, Faculty of Dentistry, Faculty of Health Sciences (Nursing, Obstetrics, Physiotherapy, Public Health, Emergency Medicine, Dietetics), Pharmaceutical Faculty, Medical Laboratory Diagnostics Faculty, and at the Radiology and Radiotherapy Department. The programs were reviewed and accepted by the Ministry of Health. The program plan mainly focused on adjuvant treatment of tumors, including radiotherapy, chemotherapy of solid tumors, oncological surgery, and practical knowledge related to prevention and early diagnosis of tumors. The oncology curriculum was based on the experience and recommendations of the European Association for Cancer Education (EACE) and the Union for International Cancer Control (UICC). The program was developed at meetings with representatives of all Polish medical universities responsible for oncology education [7, 8] (The philosophy of cancer education implemented in the curriculum is described in a separate section below.)
2. At most universities (Table 1), the number of classes devoted to oncology education was increased to 70, the postulated program minimum, and an increased number of class hours was devoted to palliative medicine (Table 1).
3. An oncology education coordinator was successfully appointed at every medical university (Table 2). The main duty of the coordinator is to preside over a team of academic teachers of basic sciences and preclinical and clinical subjects in their efforts to define a detailed program of oncology classes. The coordinator is also responsible for filling gaps in the program and for eliminating contradictions and redundancies.
4. The curriculum and the textbook served as a basis for preparing a set of test questions to ensure that the standard of the final course examination is similar at all medical universities. Additionally, all medical universities introduced a set of core lectures and seminars that were considered of key importance for oncology education. There is, however, still no final examination at a number of universities at the end of the oncology course, and students automatically receive a pass credit based only on course attendance (see Table 2).
5. An inter-university website for medical students was developed (http://www.e-onkologia.am.wroc.pl/) and contains a series of online lectures in oncology incorporating many of the basics of the uniform oncology education program. The site also includes links to oncology databases, information about the NPCND, the oncology treatment system and oncological specialties, and online tests for students (see next point).
6. Online oncology tests (“oncotests” available at http://www.e-onkologia.am.wroc.pl/onkotest.php) were developed for students to practice their knowledge. These include tests respectively for students of the following: (1) Medicine and Dentistry, (2) Health Sciences (Nursing, Obstetrics, Physiotherapy, Public Health, Emergency Medicine, Dietetics), and (3) Pharmacy and Medical Laboratory Diagnostics. The tests have been updated on several occasions. The site has been visited over 121,000 times.
7. Between 2006 and 2009, several intra- and inter-university conferences and teleconferences were arranged for program coordinators and university academic affairs representatives to discuss progress, difficulties, and challenges.
8. Oncology educational institutions were equipped with a variety of teaching aids including computers and software, audiovisual equipment, and anatomical models.
9. A modern comprehensive textbook of oncology for undergraduate students was published in 2007. It presents a multidisciplinary approach to diagnosis and treatment, with special attention given to the practical knowledge indispensable for future physicians (general practitioners).
10. Subscriptions to online foreign oncology journals were made to supplement hard-copy Polish and foreign publications.

Regrettably, it proved impossible to increase the number of integrated oncology units at Polish universities (Table 2). Most university teaching (Table 2) takes place at oncology hospitals that are not parts of medical universities, and facilities such as classrooms, libraries, and even canteens are scarce or non-existent. This can be considered a failure of the program (Table 3). Also, the number of academic teachers representing the main branches of oncology has not increased significantly (Table 3).

### Philosophy of Cancer Education in Poland Today as a Result of the Program

Public awareness of cancer in Poland remains low, as is evident from the alarmingly low participation in public screening programs (47 % in breast cancer screening, 23 % in cervical cancer screening—data retrieved August 1, 2013 from http://www.wok.wroclaw.pl/dzialalnosc-wok/statystyki-wok). Efforts are being made, therefore, to ensure that in their first years at a university, students learn about the social and medical aspects of cancer, early symptoms, basics of diagnostics, multidisciplinary treatment, and oncology patient management. Special efforts are now being undertaken to emphasize the role of cancer prevention and screening and to describe the role of epidemiology in identifying risk factors.

As a result of the program, students in the final stage of their studies now take a course integrating the various concepts and skills taught during other preclinical and clinical classes in etiology, epidemiology, prevention, diagnostics, and treatment of cancer as well as patient post-treatment review and management.

Lecturers emphasize the role of radiotherapy which is not normally discussed in other classes (in Poland, radiotherapy apparatus is usually only available in specialized oncology centers). Special importance is also given to the unique nature of solid tumor chemotherapy, oncological surgery and gynecology, and to the need for multidisciplinary therapy, with cooperation between radiotherapists, clinical oncologists, and doctors of other specialties. Moreover, lecturers urge primary and secondary prevention and cancer awareness among all doctors and other healthcare professionals and underline the pivotal role that primary care physicians play in diagnosing early stage cancer. Classes are held in cancer clinics where combination therapy is used. All practical classes are held directly at cancer patients’ beds. Multimedia presentations and anatomical models are used during seminars.

Students are involved in tumor staging and treatment planning and learn how to inform patients of the disease and of the need for long therapy that often has numerous side-effects. They are also actively involved in diagnosing, treatment, as well as analyzing test results and treatment effects. Oncology education also involves management of post-treatment complications that may be discovered by doctors of other specialties during routine examinations.

## Discussion

All doctors encounter patients with cancer, so all medical students should learn about cancer. However, a question raises: What should be learned at the undergraduate level, and what are the most appropriate teaching methods? The medical universities should be aware that only students with an interest in oncology will receive specialized oncological postgraduate training [9].

Several attempts to define core skills and competencies in oncology that medical students should possess can be mentioned. Since in 1993, the General Medical Council published a comprehensive review of medical education; medical schools in the United Kingdom have modified their undergraduate teaching [10]. The Australian Ideal Oncology Curriculum for Medical Students was developed in 1999 by the Cancer Council Australia’s Oncology Education Committee. This multidisciplinary group of cancer clinicians and educators representing all medical schools in Australia and New Zealand extensively consulted their “ideal” curriculum with the academic staff of all medical schools in Australia and New Zealand. “Cancer Council Australia recommends that the material in the Ideal Oncology Curriculum should appear somewhere in a medical course, not necessarily in a cancer block, to provide a core of knowledge about cancer for the medical graduate. It will assist in enabling the introduction of patient-centered skills simultaneously with a range of technical skills” [11].

In 2001, Barton et al. carried out a questionnaire which covered areas of knowledge and attitudes identified in the Australian Ideal Oncology Curriculum as essential for graduating medical students. They observed that although curricula introduced a new course material, it did not produce doctors with better knowledge about cancer. They pointed out that recent students have less exposure to cancer patients than those who trained 10 years ago [12]. Cave et al. carried out a questionnaire for all 5,143 newly qualified doctors in UK in 2005. They observed low levels of exposure of medical students in the UK to patients with cancer [13]. Being aware of those observations, an attempt was made in Polish curriculum and study design to hold classes in cancer clinics where combination therapy is used. All practical classes are held directly at cancer patients’ beds. We observe that the involvement of patients in teaching and learning in oncology is popular with students and improves their knowledge, attitudes, and communication skills.

Gaffan et al. [9] point out that cancer teaching can be fragmented across disciplines with resulting risk of omission or duplication of cancer skills and knowledge. In our program, an oncology education coordinator was appointed at every medical university (Table 2) to preside over academic teachers of basic sciences and preclinical and clinical subjects in their efforts to define a detailed program of oncology classes (filling “gaps” in the program). Several authors describe computer learning modules for undergraduate teaching oncology [14–19]. Although none of the authors were able to show that computer-assisted learning improved student learning, in our program, an inter-university website for medical students and online oncology tests were developed (http://www.e-onkologia.am.wroc.pl/). Several authors describe oncology courses, among them are the following: portfolio learning [20], summer schools [21], oncology module taught by cancer patients [22], summer oncology fellowship [23], and attachments in cancer and palliative care [24]. The provided observations suggest that the introduction of courses has better prepared graduating doctors to care for patients with cancer. To increase the quality of education of our students interested in oncology, we established the Students’ Scientific Societies to provide an opportunity of regular meetings, enhancing motivation for expanding the interest in oncology, promoting self-learning, expanding communications skills, teamwork, and building skills valuable in future professional career as a doctor [25].

## Conclusions

The program for improving undergraduate oncology education ran in Poland for 5 years (to 2010), and over that period, numerous positive changes in the quality of oncology training were observed. These include the implementation of a uniform course of training at medicine faculties [7, 26], an increase in the number and duration of oncology-related classes, and the introduction of a modern textbook for oncology students. Special coordinators were appointed to ensure the successful implementation of the program at every university. A constant increase in the number of classes devoted to oncology has also been observed at other faculties [27].

The biggest remaining problem of oncology in an academic setting in Poland today is that existing university oncology units are seldom integrated. Very few universities have their own facilities and patient rooms for oncology training, and most “hands-on” teaching still has to be done externally. Adult patient oncology at Polish universities is now the last and only branch of medicine that lacks a multidisciplinary approach [27].

Improving the quality of undergraduate oncology education in Poland is a long-term process. It has required the collaboration of all the authorities of each individual medical university and significant financial support to implement the individual elements of the program. Via the National Program for Combating Neoplastic Diseases, the Ministry of Health has introduced positive changes in the quality of undergraduate education in oncology by providing financial, legal, and organizational assistance.

## Abbreviations

NPCND: National Program for Combating Neoplastic Diseases
MU: Medical university
